# Inhaled methoxyflurane (Penthrox®) versus placebo for injury-associated analgesia in children—the MAGPIE trial (MEOF-002): study protocol for a randomised controlled trial

**DOI:** 10.1186/s13063-019-3511-4

**Published:** 2019-07-04

**Authors:** Stuart Hartshorn, Michael J. Barrett, Mark D. Lyttle, Sue Anne Yee, Alan T. Irvine, in collaboration with Paediatric Emergency Research in the UK and Ireland (PERUKI)

**Affiliations:** 1 Emergency Department, Birmingham Children’s Hospital, Steelhouse Lane, Birmingham, B4 6NH UK; 2 Emergency Department, Our Lady’s Children’s Hospital, Crumlin, Dublin, Ireland; 3 National Children’s Research Centre, Crumlin, Dublin, Ireland; 4 School of Medicine, University College Dublin, Dublin, Ireland; 5 Emergency Department, Bristol Royal Hospital for Children, Bristol, UK; 6 Faculty of Health and Applied Sciences, University of the West of England, Bristol, UK; 7 Medical Developments International Limited, Scoresby, VIC Australia

**Keywords:** Methoxyflurane, Penthrox®, Paediatric, Analgesia, Pain, Injury, Trauma, Randomised trial

## Abstract

**Background:**

Pain from injuries is one of the commonest symptoms in children attending emergency departments (EDs), and this is often inadequately treated in both the pre-hospital and ED settings, in part due to challenges of continual assessment and availability of easily administered analgesic options. Pain practices are therefore a key research priority, including within the field of paediatric emergency medicine. Methoxyflurane, delivered via a self-administered Penthrox® inhaler, belongs to the fluorinated hydrocarbon group of volatile anaesthetics and is unique among the group in having analgesic properties at low doses. Despite over 30 years of clinical acute analgesia use, and a large volume of evidence supporting its safety and efficacy, there is a paucity of randomised controlled trial data for Penthrox®.

**Methods:**

This is an international multi-centre randomised, double-blind, placebo-controlled phase III trial assessing the efficacy and safety of methoxyflurane delivered via the Penthrox® inhaler for the management of moderate to severe acute traumatic pain in children and young people aged 6–17 years. Following written informed consent, eligible participants are randomised to self-administer either inhaled methoxyflurane (maximum dose of 2 × 3 ml) or normal saline placebo (maximum dose 2 × 5 ml). Patients, treating clinicians and research nurses are blinded to the treatment. The primary outcome is the change in pain intensity at 15 min after the commencement of treatment, as measured by the Visual Analogue Scale (VAS) or the Wong-Baker FACES® Pain Rating scale, with the latter converted to VAS values. Secondary outcome measures include the number and proportion of responders who achieve a 30% reduction in VAS score compared to baseline, rescue medication requested, time and number of inhalations to first pain relief, global medication performance assessment by the patient, clinician and research nurse, and evaluation of adverse events experienced during treatment and during the subsequent 14 ± 2 days. The primary analysis will be by intention to treat. The total sample size is 110 randomised and treated patients per treatment arm.

**Discussion:**

The Methoxyflurane AnalGesia for Paediatric InjuriEs (MAGPIE) trial will provide efficacy and safety data for methoxyflurane administered via the Penthrox® inhaler, in children and adolescents who present to EDs with moderate to severe injury-related pain.

**Trial registration:**

EudraCT, 2016–004290-41. Registered on 11 April 2017.

ClinicalTrials.gov, NCT03215056. Registered on 12 July 2017.

**Electronic supplementary material:**

The online version of this article (10.1186/s13063-019-3511-4) contains supplementary material, which is available to authorized users.

## Background

Pain is the most common symptom in patients requiring emergency healthcare, with acute trauma frequently the cause [1, 2]. There are many short-term and long-term consequences of inadequately treated acute pain, and it is universally accepted that its management should begin at the earliest opportunity [3, 4]. In 2012, the Royal College of Emergency Medicine [5] identified pain management as the most popular indicator for quality of care in an emergency department (ED). Current standards recommend the simple and timely sequential process of pain recognition, assessment, measurement, therapeutic actions/interventions and reassessment [6]. Pain-relief regimes work optimally when effective analgesics are supported by formal protocols and guidelines underpinned by staff and patient education.

Patients with traumatic pain often receive inadequate analgesia in pre-hospital and ED settings [7, 8]. The reasons for this are multifaceted, but in children they likely include challenges such as assessment tool application, perceived competing clinical priorities, ease/route of drug administration, availability of therapeutic options and a limited evidence base. Paediatric emergency physicians have prioritised pain practices as a key research priority [9]. The ideal analgesic for acute pain should have rapid onset of action, act over an appropriate and predictable period of time, be well tolerated and be effective across a wide range of pain types in different populations. The time to clinical analgesic effect of oral or topical analgesics is too slow to meet the standards of treating trauma-related moderate or severe paediatric pain [10]. Intranasal or inhaled delivery of analgesic agents allows quick and simple drug administration without the distress of intravenous cannulation and may help improve the management of acute moderate to severe pain in children in pre-hospital and ED settings.

Methoxyflurane (2,2-dichloro-1,1-difluoro-1-methoxyethane) is a volatile fluorinated hydrocarbon [11]. It is a colourless liquid with a fruity odour. Methoxyflurane was first introduced as an inhalational anaesthetic in the 1960s. In the 1970s, due to availability of newer anaesthetic agents and case reports of dose-related renal tubular damage at high anaesthetic doses of long duration, its use was generally discontinued. However, unlike other fluorinated anaesthetics, methoxyflurane has analgesic properties at much lower doses, which are not associated with nephrotoxicity [12].

In Australia, methoxyflurane (Penthrox®) is licensed for short-term relief of acute pain in adults and children (including minor surgical procedures), and has been used for pain relief in sub-anaesthetic doses for over 30 years. The number of administrations has now exceeded 6 million, with only a single report of nephrotoxicity at recommended analgesic doses. There were confounding factors in this adult case report [13].

Methoxyflurane is available in 3-ml ampoules containing pure methoxyflurane to be used in a self-administered hand-held and portable Penthrox® inhaler, to a potential maximum recommended dose in 24 h of two 3-ml vials [14]. Methoxyflurane is absorbed rapidly, resulting in fast onset of analgesia, usually within 6–10 inhalations [14]. As a non-controlled drug, the preparation time between prescription and patient administration is quick, and Penthrox® likely requires less patient monitoring than opioid analgesics. Methoxyflurane administered via the Penthrox® inhaler may eliminate the need for opioid analgesia for dislocations or fractures, since the pain relief may be sufficient for reduction or splinting.

A literature review on the use of methoxyflurane in EDs and prehospital settings recommended that large, blinded, placebo-controlled studies investigating its analgesic efficacy should be conducted [15]. This was largely addressed in trial MEOF-001 (the STOP trial), conducted at six sites in the UK between 2011 and 2012 [16]. However, the majority of the participants were adults, with just 90 adolescent patients (aged 12–17 years) and no younger children. One other paediatric RCT showed that methoxyflurane was effective for the treatment of pain associated with upper limb fractures, but this trial included just 41 patients over the age of 5 years [17].

The Methoxyflurane AnalGesia for Paediatric InjuriEs (MAGPIE) trial—a well-powered, randomised, blinded, placebo-controlled trial—therefore aims to determine whether methoxyflurane is effective and safe in the treatment of moderate to severe injury-related pain in children and young people aged 6–17 years.

## Methods

### Aim

This trial aims to establish the efficacy and safety of methoxyflurane, when self-administered via the Penthrox® inhaler, in children and adolescents presenting to EDs with moderate to severe injury-related pain.

### Trial design

**Fig. 1:**
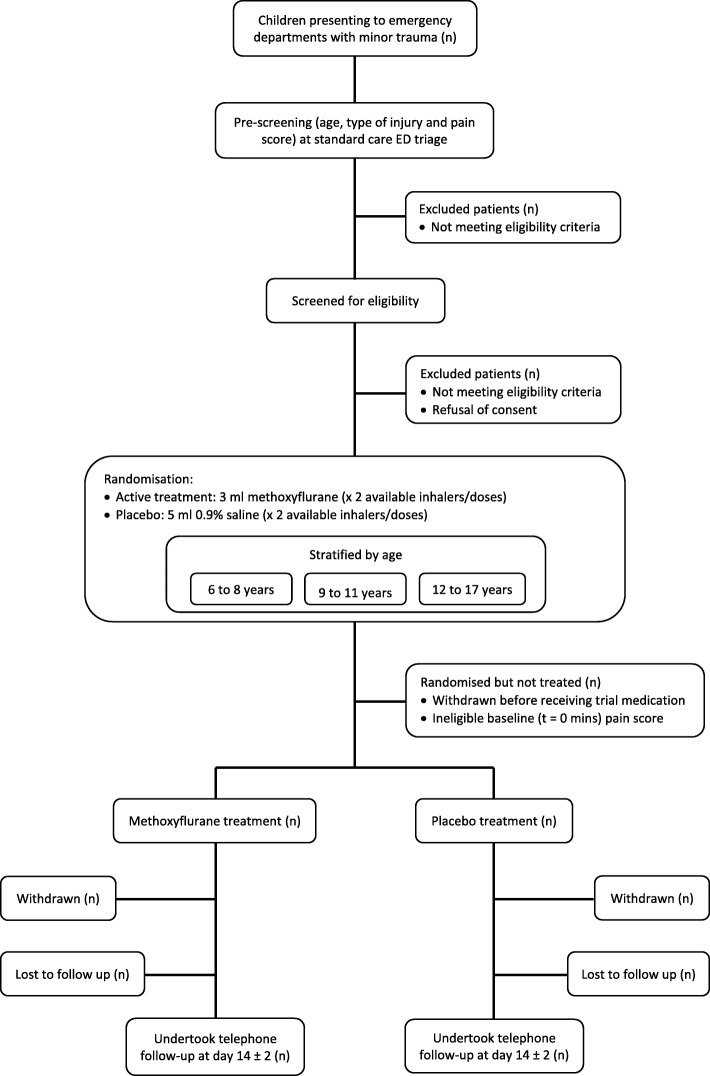
MAGPIE trial flowchart. ED emergency department, MAGPIE Methoxyflurane AnalGesia for Paediatric InjuriEs

This is an international multi-centre, randomised, double-blind, placebo-controlled, explanatory phase III trial assessing the efficacy and safety of methoxyflurane delivered via the Penthrox® inhaler for the management of moderate to severe pain associated with minor trauma. Recruitment and randomisation are stratified by age group bandings of 6–8 years, 9–11 years and 12 to < 18 years in a 2:2:1 ratio. A trial flowchart is shown in Fig. 1.

**Fig. 2:**
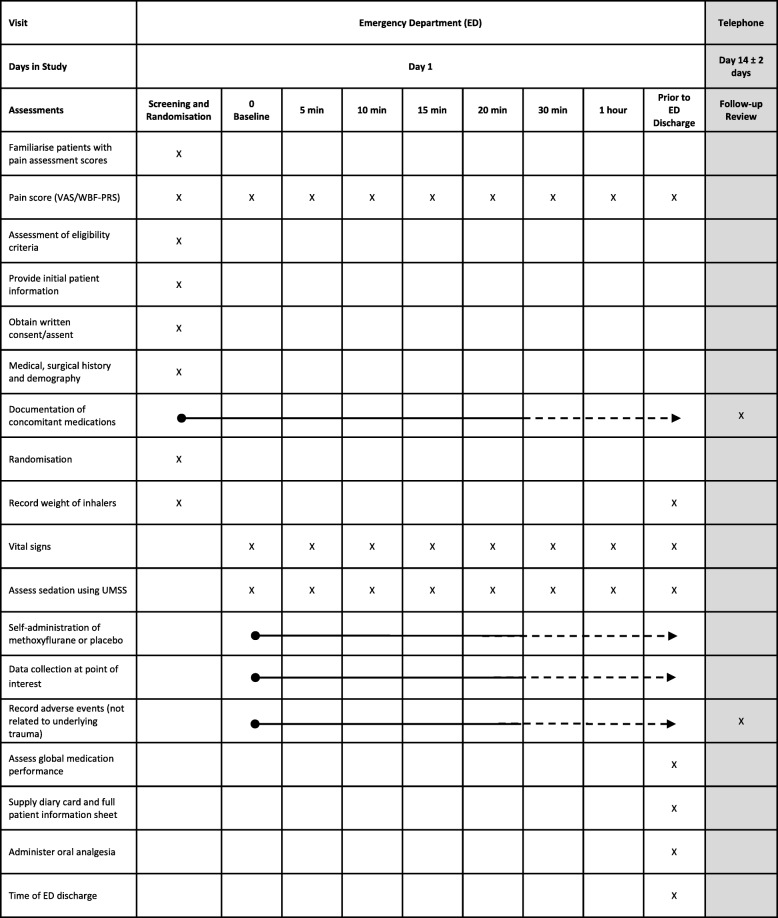
MAGPIE trial schedule of trial events. MAGPIE Methoxyflurane AnalGesia for Paediatric InjuriEs, UMSS University of Michigan Sedation Scale, VAS Visual Analogue Scale, WBF-PRS Wong-Baker FACES® Pain Rating scale

Key trial events are outlined in Fig. 2.

The protocol for this trial has been written in accordance with the Standard Protocol Items: Recommendations for Trials (SPIRIT) checklist (Additional file 1).

### Trial setting

Nine EDs within the UK and Ireland are participating, selected from the membership of Paediatric Emergency Research in the UK and Ireland (PERUKI) (www.peruki.org), a collaborative paediatric emergency medicine research network [18]. Participating sites may be tertiary or district general hospitals with EDs that treat either children alone or both children and adults. Site selection was based on several factors, including previous research experience, site research infrastructure and number of likely recruits estimated from a preparatory service evaluation.

### Trial participants

#### Inclusion criteria

The following inclusion criteria apply:

- Age ≥ 6 years to ≤ 17 years.
- Attending an ED following minor trauma.
- Pain scores (at screening and just prior to administration of study treatment) measuring between 60 and 80 mm on the Visual Analogue Scale (VAS) [19] or, where the patient is unable to understand the VAS, pain scores between 6 and 8 using the Wong-Baker FACES® Pain Rating scale (WBF-PRS).

#### Exclusion criteria

Patients are not enrolled to the trial if they meet any of the following criteria:

1. Critical, life-threatening or limb-threatening condition requiring immediate management.
2. Open fractures.
3. Any other clinical condition that may, in the opinion of the Investigator, impact the patient’s ability to participate in the trial.
4. Patient deemed not cognitively capable of effectively self-administering the trial treatment.
5. Treatment with any analgesic agent within 5 h prior to presentation to the ED, with the exception of Entonox (50% nitrous oxide and 50% oxygen mixture, which is prohibited within 30 min prior to presentation to the ED), diclofenac (which is prohibited within 8 h prior to presentation to the ED) or oral morphine (which is prohibited within 10 h prior to presentation to the ED).
6. Chronic pain.
7. Receipt of an investigational medicinal product (IMP) in the preceding 3 months.
8. Known pregnancy or breastfeeding females.
9. Personal or familial hypersensitivity to methoxyflurane or any fluorinated anaesthetics.
10. Requirement for oxygen therapy.
11. Known or genetic susceptibility to malignant hyperthermia or a history of severe adverse reactions (ARs) in either patient or relatives.
12. Clinically evident respiratory depression.
13. Previous use of methoxyflurane (including as an IMP).
14. History of signs of liver damage including after previous methoxyflurane use or halogenated hydrocarbon anaesthesia.
15. Known significant renal impairment.
16. Altered level of consciousness due to any cause including head injury, drugs or alcohol.
17. Known significant cardiovascular disease (e.g. pathological arrhythmia).
18. Inability to participate in telephone follow-up on day 14 (± 2 days).

### Primary outcome

The primary outcome is the change in VAS (including WBF-PRS scores subsequently converted to VAS values) at 15 min after the commencement of treatment.

### Secondary outcomes—efficacy

- Number and proportion of participants in each arm who achieve a 30% reduction in VAS score at 15 min.
- Number and proportion of participants in each arm who achieve a 30% reduction in VAS score at 5, 10 and 20 min.
- Change in pain intensity (VAS) at 5, 10, 15, 20 and 30 min, and every 30 min thereafter until the point of ED discharge.
- Number and proportion of participants in each arm requesting rescue medication within 20 min of start of treatment, and any time during treatment.
- Time to requesting rescue medication.
- Time to first-reported analgesic effect.
- Number of inhalations until first-reported analgesic effect.
- Whether the patient covered the dilutor hole in the inhaler during inhalation.
- Global medication performance assessment by patient, clinician and research nurse, using a 5-point Likert scale.

### Secondary outcomes—safety

- AEs experienced during treatment (not associated with the underlying trauma).
- Significant changes in physiological parameters or sedation scores during treatment.
- Evaluation of AEs at 14 ± 2 days following ED discharge using a follow-up questionnaire that includes high-output nephrotoxicity.

### Screening

Screening commences once a child is registered in the ED with minor trauma, defined as “a non-critical and non-limb threatening physical wound or injury of the tissues”; this definition includes soft tissue injuries, fractures or ligament injuries of the extremities, burns, penetration by foreign bodies, lacerations, dislocation and contusions. Trial research nurses identify and screen potentially eligible patients. The screening process includes the measurement of the screening pain score, using either the VAS or WBF-PRS, which must fall within the acceptable trial range. The unique participant screening form records reasons for non-randomisation where appropriate.

### Consent

Eligible patients and their parent or legal guardian are given sufficient time to read a summary Patient Information Sheet (PIS), approved by the Research Ethics Committee (REC), which contains all essential information regarding the MAGPIE trial. After the participant has been offered the opportunity to ask further questions and agrees to participate, informed consent is taken by a member of the research team who will remain blinded to subsequent randomisation and treatment allocation. For patients under the age of 16 years, written informed consent is sought from the parent or legal guardian, with verbal assent from the patient. Competent 16 and 17 year olds provide their own written consent. At the time of discharge from the ED, a detailed PIS is provided, which has also been approved by the REC.

### Enrolment and randomisation

Randomisation sequences, stratified by age group (6–8 years, 9–11 years and 12 to < 18 years in a 2:2:1 ratio), are prepared by an independent statistician. Patients are randomised in the ratio of 1:1 to the active (methoxyflurane) or placebo group. The IMP is packaged according to the randomisation sequence prepared by the independent statistician.

Following consent, an unblinded member of the trial team enrols the patient to the trial using the Interactive Web Response System (IWRS). At enrolment, the participant is allocated the next randomisation number in the appropriate stratum. Treatment allocation as per the randomisation scheme is maintained by allocating IMP or placebo with the next sequential patient number.

### Trial treatments

Methoxyflurane (active treatment) or normal saline (placebo) are administered by inhalation via the hand-held Penthrox® inhaler, and are self-administered by the patient under the supervision of a research nurse who is trained in administration.

#### Active treatment

A 3-ml sample of methoxyflurane is vaporised in a Penthrox® inhaler. On finishing the dose, the patient can request a second inhaler containing a further 3-ml dose. The maximum dose administered is 6 ml of methoxyflurane. The composition of methoxyflurane is detailed in Table 1.

**Table 1:** Composition of methoxyflurane

Investigational product	Function	Each 1 ml contains	Each 3 ml contains
Methoxyflurane	Active ingredient	99.9% w/w	99.9% w/w
Butylated hydroxytoluene	Antioxidant	0.01% w/w	0.01% w/w

Methoxyflurane is supplied as 3 ml of liquid in glass bottles in the IMP pack, and is stored below 30 °C.

#### Placebo

A 5-ml sample of commercially available sterile 0.9% sodium chloride (normal saline) for injection is loaded into the Penthrox® inhaler. The patient can request a second inhaler containing a further 5-ml dose. The maximum dose administered does not exceed 10 ml.

#### Penthrox® inhaler

**Fig. 3:**
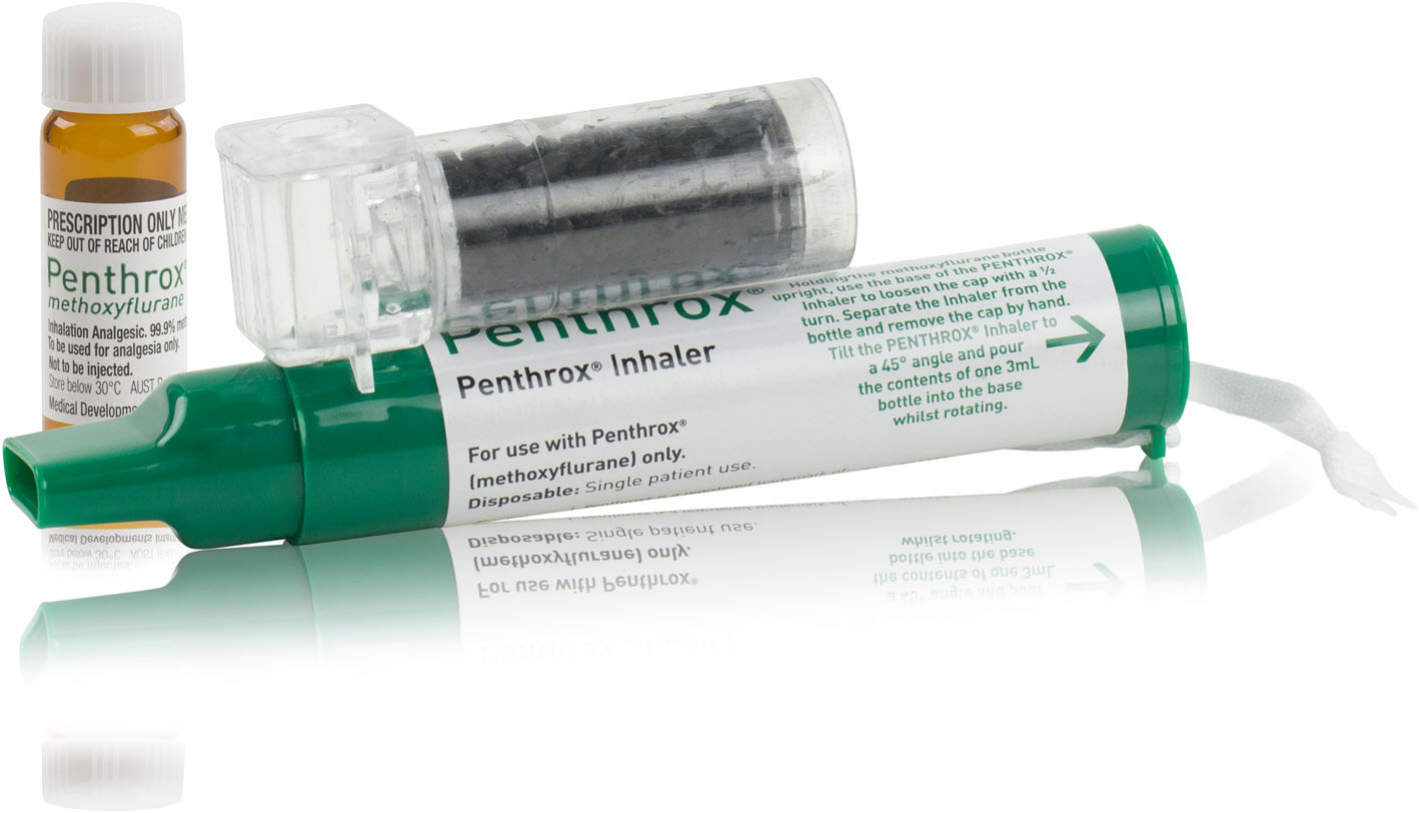
The Penthrox® inhaler

The hand-held Penthrox® inhaler is a small, lightweight, disposable, cylindrical polyethylene device, approximately 15 cm long in a distinctive green colour, shaped like a large (green) whistle (Fig. 3).

There is a dilutor hole at the mouthpiece end, which, when covered with the patient’s index finger, allows a higher concentration of treatment to be inhaled. The device contains a polypropylene S-shaped wick, which absorbs the liquid. An internal one-way valve allows air and vapour to be inhaled; this closes on expiration to prevent exhalations (containing a mixture of air, methoxyflurane and carbon dioxide) passing back through the wick. The inhaler is a single-patient use device, which prevents cross-contamination between patients, and can be easily disposed of after use.

To minimise environmental release of methoxyflurane, an activated carbon (AC) chamber is inserted into the aperture at the top of the inhaler. The AC adsorbs the exhaled methoxyflurane without causing any resistance to inhalation or expiration.

### Loading of the Penthrox® inhaler and blinding procedures

The unblinded investigator is responsible for loading and dispensing Penthrox® inhalers in such a way as to maintain the blinding for the treating clinician and the blinded research nurse. The key challenges for maintaining the blinding in the MAGPIE trial are the ability to distinguish between active IMP and placebo by the appearance of the container, volume of liquid administered and smell of methoxyflurane. The inhaler loading occurs in a closed area which is geographically separated both from the participant’s treatment area and the location of blinded research staff.

Each patient is allocated a pack containing two Penthrox® inhalers, each with an AC chamber attached, two containers of either methoxyflurane or sterile normal saline and four sealable plastic bags. Placebo packs include a container of methoxyflurane which is used for blinding (see next section). At enrolment, the assigned participant number (linked to the randomisation number) is entered on all pack labels. Packs are prepared by an independent pharmaceutical distributor.

Each Penthrox® inhaler is loaded by pouring the full contents of either one 3-ml bottle of methoxyflurane (active treatment) or one 5-ml ampoule of sterile normal saline (placebo) into the inhaler, in accordance with the treatment allocation of the patient. Different volumes of methoxyflurane and normal sodium chloride are used to minimise the weight difference because of the significant solution density difference between methoxyflurane and 0.9% sodium chloride.

The inhaler is placed in a plastic bag, which is sealed and weighed prior to being dispensed to blinded investigators. Before sealing the plastic bag containing a placebo inhaler, several drops of methoxyflurane are placed on the wristband of the placebo inhaler, so that it is not possible for participants or blinded investigators to distinguish between active IMP and placebo based on a masking smell upon opening the plastic bag.

A second identical inhaler is prepared using the same treatment allocation. This second inhaler is to be available if requested by the patient if they deem the first inhaler is no longer effective. The maximum dose administered does not exceed 6 ml of methoxyflurane (2 × 3 ml) or 10 ml of placebo (2 × 5 ml).

Allocation of investigators to blinded or unblinded processes is the responsibility of the site Principal Investigators; once allocated, these investigators may not switch roles for the duration of the trial.

### Administration of trial treatments

The research nurse provides training in the use of the device to the patient using a demonstration inhaler which contains no IMP and supports them in its use as necessary for the duration of IMP administration. Baseline vital signs (heart rate, blood pressure, respiratory rate and oxygen saturation) are measured along with an assessment of baseline sedation level using the University of Michigan Sedation Scale (UMSS) [20].

After randomisation, the baseline (time = 0) pain score is assessed, to ensure relative stability of pain intensity. If the baseline pain score has fallen outside the range of 60–80 mm on the VAS or 6–8 on the WBF-PRS, the patient is withdrawn from the trial (randomised but not treated).

If successfully randomised, the research nurse assists the participant to self-administer 10 successive inhalations of the IMP. Participants and parents/legal guardians are advised that rescue analgesia is available immediately on request at any time. Permitted rescue medications are intranasal fentanyl, intranasal diamorphine, intranasal ketamine, intravenous morphine, oral morphine or Entonox (50% nitrous oxide and 50% oxygen mixture), dependent on the standard practice of the participating site and at the discretion of the treating clinician. This approach is in line with current recommended practice [10] for the management of moderate to severe pain. Standard-of-care oral analgesic medications (paracetamol and ibuprofen) may be administered as concomitant medications alongside rescue medication. Participants who report pain relief due to IMP inhalation are advised to continue to self-administer additional inhalations, as required, during their ED attendance.

### Trial assessments

Participants are evaluated according to the schedule of events outlined in Fig. 1.

#### Pain scores

Pain intensity is re-assessed using the VAS or WBF-PRS at 5, 10, 15, 20 and 30 min after the start of inhalation of the trial treatment, and every 30 min thereafter until ED discharge. Additional unscheduled pain scores are assessed at the time of any other key events, such as during any injury treatment, prior to rescue medication administration, prior to premature trial withdrawal or just prior to ED discharge.

#### Vital signs and sedation scores

Vital signs (heart rate, blood pressure, respiratory rate and oxygen saturation) and UMSS are re-assessed at 5, 10, 15, 20 and 30 min after the start of inhalation of the trial treatment, and every 30 min thereafter until ED discharge.

#### Other key events

The following events are recorded if and when they occur:

- Treatment of the patient’s injury, and the start and end times of such treatment.
- Timing and nature of any AEs (other than those associated with the underlying minor trauma) and any associated change in trial treatment administration.
- Concomitant medication administration.

#### Global medication performance

Following completion of trial treatment administration, the patient, research nurse and treating clinician are each asked to rate the global medication performance on a 5-point Likert scale (poor, fair, good, very good, excellent).

### Events at the point of ED discharge

Upon completion of trial treatment administration, all inhalers are collected, placed and sealed in the plastic bags and returned to the unblinded investigator, who weighs the inhalers inside their sealed plastic bags and records the weights following trial treatment administration.

At the point of discharge, a final pain score and vital signs are recorded. For patients requiring inpatient admission, the time of such a decision to admit should be considered the discharge time in terms of trial activity. The final injury diagnosis is documented. A single dose of oral analgesia (paracetamol and ibuprofen) is offered to all patients unless these have previously been administered or are contra-indicated. In the case of patients who are being discharged home, rather than admitted, instructions are provided to the patient/parent on suitable analgesia post discharge (as per standard practice of participating sites). The patient/parent is provided with a diary card and asked to record all concomitant medications and any symptoms experienced from the time of discharge until the follow-up telephone call. The patient/parent is advised to contact the named trial doctor or research nurse in the event of any significant adverse event (SAE), using the contact information provided on the PIS. In any cases of SAE, the patient is recalled to the site for further assessment.

### Follow-up

At 14 ± 2 days after discharge from the ED, the research nurse telephones the patient/parent for safety follow-up. A set questionnaire is used, and the patient/parent is asked to refer to the information collated on their diary card. The following points are covered during the telephone call:

- Any concomitant medication(s) (name and dose of medication) taken since discharge.
- Any AE, other than those related to the original injury.
- Presence or absence of specific symptoms and signs, including those relating to potential nephrotoxicity and hepatotoxicity.
- In any cases of significant AE, the patient is recalled to the site for further assessment.

At least three attempts are made to contact the patient to collect this follow-up information. If the three attempts are unsuccessful, the patient’s general practitioner is contacted for information on any recent medical consultations.

### Data collection and management

A paper Screening Form and a Data Collection Proforma (DCP) are used to capture trial data in real time. Patient identification on the DCP is through their unique trial number, allocated at the time of enrolment. The DCP becomes the paper source document; however, it is used in conjunction with the usual hospital practice for recording medical information.

On completion of ED trial processes, participant data are recorded via the InForm Electronic Data Capture system using an electronic Case Report Form (eCRF). These data are transcribed by the research nurse or a delegated data manager from the DCP onto the eCRF. To ensure accurate, complete and reliable data are collected, IQVIA™ (formerly QuintilesIMS™ Holdings, Inc.), the Contract Research Organisation for the MAGPIE trial, provides training to site staff in the form of investigator meetings, site initiation visits or teleconference training sessions. The data are validated, and discrepancy reports are generated following data entry to identify discrepancies, such as out of range values, inconsistencies or protocol deviations based on data validation checks programmed into the eCRF. A full electronic audit trail is created for data entry and any subsequent amendments. Prior to submission, each completed eCRF must be reviewed for accuracy by the Principal Investigator at the site, corrected as necessary and then approved. The Investigator’s e-signature serves to attest that the information contained on the eCRFs has been reviewed by the Investigator and is true and accurate. The monitor will review the eCRFs and evaluate them for completeness and consistency. The eCRF is compared with the source documents to ensure that there are no discrepancies between critical data.

The sites, the trial database and trial documentation are subject to quality assurance audit during the course of the trial by the sponsor or IQVIA™ on behalf of the sponsor. In addition, inspections may be conducted by regulatory bodies at their discretion.

### Statistical considerations

Statistical analyses are performed by IQVIA™ using SAS® version 9.4 or higher (SAS Institute, Cary, NC, USA). Any change to the data analysis methods will be mentioned in the statistical analysis plan. Any additional analysis, and the justification for making the change, will be described in the Clinical Trial Report.

#### Sample size estimation

The sample size calculations assume a treatment difference to placebo of 12.4 mm in adolescents (based on interim 15-min data from the MEOF-001 trial [16]) and 14 mm in younger children, leading to a combined overall difference to be detected of 13.3 mm. The expected variability is higher in younger children (standard deviation (SD) 28 mm) than in adolescents (SD 22.5 mm), resulting in a pooled SD of 27.1 mm.

A sample size of 110 evaluable patients per treatment arm provides at least 95% power to detect a treatment difference of 13.3 mm in change from baseline of VAS pain score after 15 min with a pooled SD of 27.1 mm. Approximately 220 patients will be randomised and treated, and the drop-out rate is expected to be minimal. This sample size will allow an overall power of the trial to deliver statistically significant results for the complete trial population.

The trial population will include a minimum of 88 children (40%) aged 6–8 years, 88 children (40%) aged 9–11 years and 44 adolescents (20%) aged 12–17 years. An exploratory subgroup analysis per age range will be conducted, but this is not expected to be sufficiently powered to demonstrate statistical significance.

#### Statistical analysis plan

The primary analysis shall be an intention-to-treat (ITT) analysis of the difference between methoxyflurane and placebo on the VAS, to include all patients who are randomised and receive trial treatment and undergo at least one post-baseline efficacy assessment. A pragmatic conversion of WBF-PRS scores to VAS scores (e.g. WBF-PRS 2 = VAS 20 mm, WBF-PRS 4 = VAS 40 mm, etc.) will be completed. The primary model will be an analysis of covariance (ANCOVA) of the VAS at 15 min following the start of inhalation of trial treatment with baseline pain as covariate, treatment and age group as fixed effect and site as random effect using the ITT population. The treatment effect shall be estimated as the average difference between the methoxyflurane-treated group and the placebo group at 15 min. Pain scores taken after the initiation of a rescue medication will be included in the primary analysis. This approach is conservative for the primary analysis and allows for patients with missing data at any time point to be included in the analysis with less bias than if patients are dropped from the analysis. Sensitivity analysis of primary endpoint pain values shall be performed using Worst Observation Carried Forward (WOCF). To exclude the possibility that conversion of any WBF-PRS scores (ordinal scale) into VAS scores (continuous scale) significantly influences the outcome of the trial, sensitivity analysis using a non-parametric test (Wilcoxon rank-sum test) will be performed.

Various methods will be used for secondary endpoint analyses. For treatment comparison of change from baseline for continuous variables with more than one measurement within a time period, repeated-measures analysis employing restricted maximum likelihood (REML)-based mixed-model repeated measures (MMRM) will be used. For continuous variables with baseline value and one post-baseline measurement, ANCOVA will be used, unless specified otherwise. Least square means (LSmeans), SE, LSmeans difference, 95% confidence interval (CI) and *p*-value will be presented. Logistic regression analyses adjusted for baseline will be performed for the binary variables, and the odds ratio and 95% CI for the odds ratio of treatment group comparisons will be given.

Analysis of safety will be for all randomised and treated patients, and according to the treatment actually received. Any protocol deviations will be defined and classified as major (i.e. affecting the primary endpoint) or minor before database lock and unblinding.

Demographic and baseline characteristics will be recorded prior to randomisation, and will be summarised by treatment group for all patients who are randomised and received trial treatment. Overall summaries will include descriptive statistics for continuous measures (number of observations, mean, SD, median, minimum and maximum) and for categorical measures (sample size and frequency).

#### Interim analyses

A blinded interim analysis is planned to verify the sample size calculation and assumptions; if necessary, the sample size will be modified. This analysis will be performed as soon as the trial has been completed by at least 30 patients in the age groups 6–8 years and 9–11 years and at least 15 adolescents. As this is a blinded interim analysis, no adjustment of the significance level is required.

### Trial monitoring

The Sponsor has engaged the services of IQVIA™, the Contract Research Organisation for the MAGPIE trial, to perform all monitoring functions within the MAGPIE trial. The monitors work in accordance with the standard operating procedures of IQVIA™, and have the same rights and responsibilities as monitors from the Sponsor. Monitoring visits are conducted according to all applicable regulatory requirements and standards. IQVIA™ is responsible for coordinating investigator meetings, investigator teleconferences and for communicating important protocol modifications to sites, trial registries and regulators.

### Safety monitoring

An independent Data Safety Monitoring Board (DSMB) is established for the trial. The DSMB is an independent group of experts who will review ARs reported during this trial. The DSMB comprises at least three members, including at least one clinician and one statistician. This board will meet at least once during the trial and additionally in the event of a SAE where the event is associated with methoxyflurane administration.

Definitions for AE, AR, SAE and suspected unexpected serious adverse reactions (SUSAR) of the Clinical Trials Regulation (EU No. 536/2014), based on the principles of Good Clinical Practice, apply to the MAGPIE trial. The AE reporting period begins from the point of written consent until day 14 (with an allowed window period of 2 days for the telephone follow-up call to be performed). The Principal Investigator, or designee, records all directly observed AEs and all AEs reported by the patient/parent during the follow-up telephone call. The Principal Investigator, or designee, assesses all AEs for seriousness, causality and severity and, if the AE is related to the trial treatment, for expectedness. AEs are assessed as unrelated, unlikely, possibly, probably or definitely related to the trial treatment, to the Penthrox® inhaler device and to any trial procedure. All AEs will be followed up to resolution, meaning that the patient has returned to a baseline state of health or the Investigator does not expect any further improvement or worsening of the AE.

Any SAE is reported immediately (or no later than 24 h after site staff becoming aware of it), using the electronic SAE report form within the eCRF. This generates an automatic email alert to the Sponsor’s pharmacovigilance provider. As a backup, all SAEs should be reported by fax/email to the Sponsor’s pharmacovigilance provider using the SAE paper report form, no later than 24 h after site staff becoming aware of it. The SAE report will contain as much available information concerning the SAE to enable the Sponsor (or an authorised representative) to file a report, which satisfies regulatory reporting requirements.

The Sponsor’s pharmacovigilance provider is responsible for reporting all SUSARs and any other applicable SAEs to regulatory authorities, ethics committees and Investigators, in accordance with national regulations. This will occur within 7 days for fatal and life-threatening events and 15 days for other SUSARs, unless otherwise required by national regulations. The Sponsor’s designee will also prepare an expedited report for other safety issues where applicable.

### Emergency unblinding

Blinded investigators are strongly discouraged from requesting the blinding be broken for an individual patient, unless there is a patient safety issue that requires unblinding and would change patient management. The process for unblinding is handled through the IWRS which is accessible at all times. The investigator should inform the Sponsor or IQVIA™ prior to unblinding if possible, or as soon as possible afterwards.

### Confidentiality

The patient trial number is recorded on all documents related to the trial. All personal information, including patient name, is removed or rendered illegible from any supportive documentation submitted with the eCRF, such as laboratory or hospital records. Participant medical information is confidential and disclosure to third parties is prohibited.

The Sponsor, or designee and auditor, may access patient records for the purpose of monitoring this trial, auditing and managing progress details. Patient records will only be accessed in secure facilities within the site in order to check the information and verify the clinical trial procedures, whilst maintaining patient confidentiality.

### Dissemination

The results of the MAGPIE trial will be reported in a clinical trial report, written in accordance with the International Council for Harmonisation of Technical Requirements for Pharmaceuticals for Human Use E3 guidelines, and will be submitted in accordance with local regulations. Results will also be published in a peer-reviewed journal or journals. Publications will be distributed to participating centres, and throughout relevant networks including PERUKI and other international paediatric emergency medicine networks. Authorship will be granted based on scientific input and recruitment efforts and will be granted upon decision of a publication committee. This committee will include, among others, the Chief Investigator and the Sponsor. Findings will also be presented at relevant national and international scientific (e.g. emergency medicine, paediatric, pain) conferences. Raw data will remain the intellectual property of the Sponsor and are not currently planned for release.

## Discussion

The MAGPIE trial presents a number of challenges which were carefully considered during the initial set-up phase and at the initial and subsequent investigator meetings.

### Challenges and opportunities for paediatric emergency research

Despite conducting a thorough and considered process for the selection of sites, several elements of the MAGPIE trial are novel for many of the participating sites. The trial demands efficient working in the early stages of the patient journey, requiring quite complex organisational processes to be well thought out and constructed. To date, successful recruitment has been achieved via close communication between the participating sites, IQVIA™ and the Sponsor, with leadership from PERUKI, providing frequent opportunities to share best practice in overcoming any obstacles to trial delivery. Specific examples include weekly recruitment emails, quarterly newsletters, investigator teleconferences and face-to-face investigator meetings. For many participating EDs, the MAGPIE trial represents their first exposure to commercial research, and is hence breaking new ground for the PERUKI network.

### Minimising delay to analgesia

The necessity of acquiring trial consent whilst simultaneously preventing or minimising any delay to analgesia for patients who are in acute moderate to severe pain is a considerable practical, administrative and ethical challenge. It is essential to ensure successful trial site engagement by optimising the efficiency of trial activity, particularly in the early screening and enrolment stages, to prevent or minimise delays to appropriate analgesia administration. Sites identify potential patients for screening as soon as possible after ED arrival. Where feasible, the preferred model is for dedicated research nurses to identify patients immediately after registration using the ED electronic patient management system, and to assess them immediately, thus removing the necessity to wait in the standard queue for initial nurse assessment/triage. For patients who meet the eligibility criteria, the written information provided takes the form of an initial brief summary sheet which contains all of the essential trial information.

### Placebo control in the context of moderate to severe pain

Half of the patients recruited to the MAGPIE trial will receive trial treatment in the form of placebo. During the consent process and during the trial training provided by the research nurse, it is emphasised that rescue analgesia is available at any time on request. Furthermore, the treating blinded clinician is trained to assess the patient at the 20-min time point and to consider the need for rescue analgesia if the pain level has not decreased. The research nurse ensures that, before a patient is enrolled, there is a clinician who will be immediately available to prescribe rescue analgesia or, where possible, that the rescue analgesia is drawn up in parallel with the study treatment.

### Maintaining blinding

The time-critical subject of this trial and the properties of methoxyflurane mean that the Penthrox® inhalers cannot be loaded within a pharmacy clinical trials unit. For this to occur within the ED, it is essential that several members of the ED and research teams contribute to the trial, working in pre-designated blinded and unblinded teams. Participating sites have each identified a preparation area for the loading of the inhalers which is separate from the standard clinical area. It is within this preparation area that the unblinded investigators add the drops of methoxyflurane onto the wristbands of the placebo inhalers, which provides the masking smell to prevent the participants and blinded investigators from distinguishing between active IMP and placebo. No cross-over between blinded and unblinded teams is permitted for the duration of the trial.

## Trial status

The concept and framework of the trial was originally presented as a Paediatric Investigation Plan to the Paediatric Committee (PDCO) of the European Medicine’s Agency for review and approval, and this informed the subsequent protocol design.

Recruitment started in July 2017 using a previous version of the MEOF-002 protocol (version 3.0, 24 May 2017).

A substantial amendment (amendment number “Protocol V4”) for the current protocol MEOF-002 version 4.0 (22 June 2018) was reviewed and approved by the following bodies:

- Research governance: Health Research Authority (HRA) and Health and Care Research Wales (HCRW) on 25 September 2018 (reference IRAS 220282) and Our Lady’s Children’s Hospital, Crumlin, Ireland on 31 October 2018 (GEN/539/17).
- Medicine regulators: Medicines & Healthcare products Regulatory Agency (MHRA) on 21 September 2018 (reference 33,389/0002/001–0004) and The Health Protection Regulatory Authority (HPRA), Ireland on 28 September 2018 (reference 2,187,763).

The key changes within this amendment were as follows:

- Addition of Entonox (50% nitrous oxide and 50% oxygen mixture) to the list of permitted pre-hospital analgesics, provided it was administered at least 30 min prior to ED arrival. This was in response to feedback from sites, in order to reduce exclusion of patients who were otherwise eligible for recruitment.
- Protocol V3.0 included two screening pain assessments 5 min apart (measured using the VAS or WBF-PRS), The first of these was replaced in Protocol V4.0 with a “pre-screening” pain assessment using whichever pain tool is standard practice at each individual site. This removes the need for three pain score assessments prior to randomisation for those patients who follow the default triage process within their ED.
- Clarification that, for patients requiring inpatient admission, the time of such decision to admit should be considered the discharge time in terms of trial activity.
- Following feedback from sites on their standard of care analgesic agents, the list of permitted rescue medications was expanded to also include intranasal ketamine and Entonox (50% nitrous oxide and 50% oxygen mixture).
- Protocol V3.0 allowed for standard of care oral analgesic medications (paracetamol and ibuprofen) to be given only at the time of ED discharge. V4.0 also permits these medications:
  - as concomitant medications alongside rescue medication; and
  - if there is a prolonged ED stay prior to discharge or inpatient admission, provided administration of trial treatment has ended.

All other changes were minor clarifications for any inconsistencies between different sections of the protocol. Recruitment adhering to protocol V4.0 is ongoing at the time of manuscript submission.

At the start of February 2019, 117 patients have been randomised to the trial, 96 of these have received trial treatment and nine sites are currently open to recruitment. Recruitment is scheduled to finish in July 2021.

## Additional file

Additional file 1: SPIRIT 2013 Checklist: Recommended items to address in a clinical trial protocol and related documents. (DOCX 60 kb)

## Data Availability

Not applicable.

## Abbreviations

AC: Activated carbon
AE: Adverse event
ANCOVA: Analysis of covariance
AR: Adverse reaction
DCP: Data collection proforma
DSMB: Data Safety Monitoring Board
eCRF: Electronic case report form
ED: Emergency department
HCRW: Health and Care Research Wales
HRA: Health Research Authority
IMP: Investigational medicinal product
ITT: Intention to treat
IWRS: Interactive Web Response System
LSmeans: Least square means
MHRA: Medicines & Healthcare products Regulatory Agency
MMRM: Mixed-model repeated measures
PDCO: Paediatric Committee of the European Medicine’s Agency
PERUKI: Paediatric Emergency Research in the UK and Ireland
PIS: Patient Information Sheet
REC: Research Ethics Committee
REML: Restricted maximum likelihood
SAE: Serious adverse event
SAS: Statistical analysis system
SD: Standard deviation
SUSAR: Suspected unexpected serious adverse reactions
UMSS: University of Michigan sedation scale
VAS: Visual analogue scale
WBF-PRS: Wong-Baker FACES® Pain Rating scale
WOCF: Worst observation carried forward
